# Effects of omega-3 supplementation on endothelial function, vascular structure, and metabolic parameters in adolescents with type 1 diabetes mellitus: A randomized clinical trial

**DOI:** 10.3389/fnut.2022.962773

**Published:** 2022-07-22

**Authors:** Masoud Khorshidi, Aliakbar Sayyari, Naheed Aryaeian, Beheshteh Olang, Mohammadreza Alaei, Mitra Khalili, Amirhossein Hosseini, Masoud Salehi

**Affiliations:** ^1^Department of Nutrition, School of Public Health, Iran University of Medical Sciences (IUMS), Tehran, Iran; ^2^Pediatric Gastroenterology, Hepatology and Nutrition Research Center, Research Institute for Children's Health, Shahid Beheshti University of Medical Sciences, Tehran, Iran; ^3^Children Emergency Department Karolinska University Hospital, Stockholm, Sweden; ^4^Department of Pediatric Endocrinology, Shahid Beheshti University of Medical Sciences, Tehran, Iran; ^5^Department of Radiology, Shahid Beheshti University of Medical Sciences, Tehran, Iran; ^6^Department of Biostatistics, School of Public Health, Iran University of Medical Sciences, Tehran, Iran

**Keywords:** omega-3, endothelial function, type 1 diabetes mellitus, vascular structure, triglycerides

## Abstract

**Background:**

Vascular dysfunction is a major complication of diabetes mellitus that leads to cardiovascular disease (CVD). This study aimed to examine the effects of omega-3 consumption on endothelial function, vascular structure, and metabolic parameters in adolescents with type 1 diabetes mellitus (T1DM).

**Methods:**

In this randomized, double-blind, placebo-controlled clinical trial, 51 adolescents (10–18 years) with T1DM completed the study. Patients received 600 mg/day [containing 180 mg eicosapentaenoic acid (EPA) and 120 mg docosahexaenoic acid (DHA)] of omega-3 or placebo for 12 weeks. Flow-mediated dilation (FMD), carotid intima-media thickness (CIMT), high-sensitivity C-reactive protein (hs-CRP), erythrocyte sedimentation rate (ESR), triglycerides (TG), low-density lipoprotein (LDL), high-density lipoprotein (HDL), total cholesterol, blood urea nitrogen (BUN), creatinine, fasting blood sugar (FBS), hemoglobin A1C (HbA1c), homeostatic model assessment for insulin resistance (HOMA-IR), quantitative insulin sensitivity check index (QUICKI), serum insulin (SI), urine albumin-creatinine ratio (uACR), blood pressure, and anthropometric indices were assessed at the baseline and after the intervention.

**Results:**

Following supplementation, omega-3 significantly increased FMD (3.1 ± 4.2 vs. −0.6 ± 4%, *p* = 0.006) and decreased TG (−7.4 ± 10.7 vs. −0.1 ± 13.1 mg/dl, *p* = 0.022) in comparison with the placebo group. However, no significant difference was observed regarding CIMT (-0.005 ± 0.036 vs. 0.003 ± 0.021 mm, *p* = 0.33). Although hs-CRP was significantly decreased within the omega-3 group (*p* = 0.031); however, no significant change was observed compared to placebo group (*p* = 0.221). Omega-3 supplementation had no significant effect on other variables.

**Conclusion:**

Given the elevation in FMD and reduction in TG, omega-3 supplementation can improve vascular function and may reduce the risk of cardiovascular disease in adolescents with T1DM patients.

## Introduction

Type 1 diabetes mellitus (T1DM) is a chronic autoimmune disorder characterized by insulin deficiency caused by the destruction of pancreatic β-cells (1). Based on recent epidemiological evidence, the prevalence of T1DM was 9.5% globally (2). Also, the incidence rate of T1DM is increasing annually by 2–3% worldwide (3). Children younger than 15 years possess the greatest increase in the incidence rate of T1DM (4). Current evidence suggests that genetic, environmental, and immunologic factors have crucial roles in the pathogenesis of T1DM, however, there are still some gaps in current knowledge (5). Impaired blood sugar control in patients with T1DM leads to structural and functional damage to the cardiovascular system, these factors predict an increased risk of cardiovascular disease (CVD) in adulthood, and studies have shown that more than 35% of people with T1DM develop vascular endothelial dysfunction after 5 years (6, 7). Despite the discovery of insulin in 1921 as the main treatment for T1DM, the disease continues to be correlated with substantial medical complications associated with vascular dysfunction including retinopathy, neuropathy, nephropathy, atherosclerosis, and thrombosis in the heart, peripheral arteries, and brain. The lifetime economic burden of T1DM represents a high burden of the disease on health care systems. Thus, developing new therapeutic strategies for the management of T1DM and its related complications is pivotal (8).

Vascular endothelial dysfunction is caused by decreased bioavailability of vasodilators, and one of the factors used to assess endothelial function is flow-mediated dilation (FMD), which has been shown to be impaired in adolescents with type 1 diabetes and predicts vascular disease in adulthood (9). End products of advanced glycation (AGEs), which are formed by constant exposure to high blood sugar, are one of the main factors in increasing vascular wall thickness such as carotid intima-media thickness (CIMT) in patients with diabetes; on the other hand, Ox-low-density lipoprotein (LDL), reduced bioavailability of nitric oxide (NO), and impaired endothelial NO synthase impair endothelial function (10). According to the American Heart Association, children and adolescents with T1DM and type 2 diabetes mellitus (T2DM) are at high risk for CVD in adulthood (11). However, exploring novel adjuvant therapy for the management of T1DM complications is taken into consideration by researchers.

Omega-3 fatty acids are proposed to exert favorable effects on T1DM (12). Omega-3 fatty acids are polyunsaturated fatty acids including eicosapentaenoic acid (EPA) and docosahexaenoic acid (DHA) and are found in oily fish and the liver of white fish. It possesses multiple biological properties including antioxidant, anti-inflammatory, immunomodulatory, antitumor, antidepressant, antihypertensive, and lipid-lowering effects (13). Moreover, a growing body of evidence revealed that various kinds of metabolic disorders underlying the development of diabetes were ameliorated by omega-3 administration (14). Omega-3 fatty acids can also be effective in improving vascular endothelial function by reducing inflammatory cytokines and increasing NO and oxylipins production (15, 16). It has been also shown that controlling blood pressure and lipids has greater beneficial impacts on the prevention of cardiovascular complications than controlling blood glucose, while both are necessary to be monitored (17). Some studies have been conducted to date investigating the effects of omega-3 fatty acids on the endothelial function and vascular structure in adults with different diseases and the results are contradictory (18, 19). There is no randomized clinical trial assessing the effects of omega-3 supplementation in children and adolescents with T1DM. Hence, the present clinical study aims to examine the effects of omega-3 supplementation on endothelial function, vascular structure, and metabolic parameters in adolescents with T1DM.

## Materials and methods

### Study design and participants

**Figure 1:**
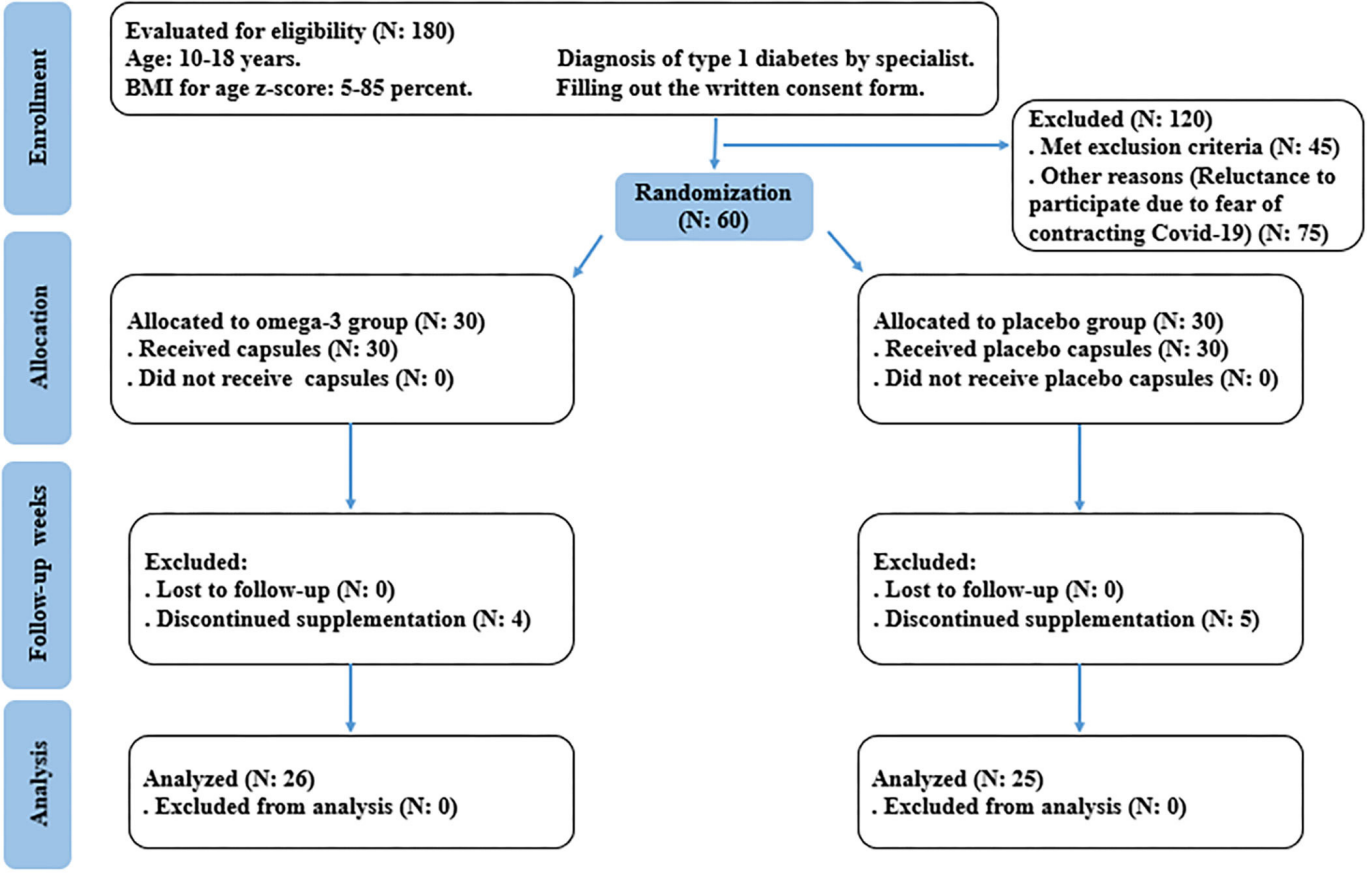
Summary of study based on the consolidated standards of reporting trials (CONSORT) flow diagram.

This was a randomized, double-blind, placebo-controlled, clinical trial. In the protocol, adolescents with T1DM, who were diagnosed by a specialist, were recruited from Mofid Children's Hospital, Tehran, Iran (20). Patients that satisfied the following inclusion and exclusion criteria were eligible to participate in this research. The inclusion criteria were considered as follows: adolescents aged between 10 and 18 years, whose body mass index (BMI) for age Z-score ranged between 5 and 85 percent, diagnosed with T1DM by a specialist, receiving insulin therapy, and at least 5 years have passed since the diagnosis of T1DM. The exclusion criteria include pregnant or breastfeeding subjects, those afflicted by endocrine and metabolic complications other than T1DM, any acute disorders, and patients who had diabetic ketoacidosis or hypoglycemia (blood sugar level lower than 50 mg/dl) in the last 12 and 3 months, respectively. In addition, subjects who consumed antihypertensive, anti-inflammatory, anticoagulant, lipid-lowering, weight-lowering agents, antioxidant, and omega-3 supplements in the last 6 months were excluded. Patients with fish and seafood allergies and smokers were also excluded. The study flow chart of enrolment, allocation, intervention, and assessment was presented in Figure 1. Written informed consent was taken from all participants or their legal guardians before contribution to the research. This investigation was approved by the Medical Ethics Committee of Iran University of Medical Sciences (IR.IUMS.REC.1400.070) and also registered on the Iranian Registry of Clinical Trials website (identifier: IRCT20210419051010N1).

### Sample size calculation

Considering a type I error of 5%, a power of 90%, and the changes in FMD values as one of the primary outcomes (21), the sample size was computed to be 25 for each study group based on the two-sided *t*-test. To compensate for an approximate attrition rate of 20% throughout the research, we increased the final sample size to 30 adolescents in each group.

### Randomization and intervention

After obtaining the informed consent eligible subjects (*n* = 60) were divided into two equal groups, stratified by sex and the amount of daily insulin intake using stratified block randomization, with a 1:1 allocation ratio. The block randomization was conducted by an assistant and the intervention allocation was blinded for both the investigators and participants. The manufacturer who was responsible for preparing the supplements was assigned a code.

A total of 60 eligible adolescents with T1DM were randomly assigned to the omega-3 (*n* = 30) and placebo groups (*n* = 30). Omega-3 capsules (600 mg) and identical placebo capsules were respectively taken by patients in the intervention and control group once a day, for 12 weeks. Karen Pharma and Food Supplement company provided omega-3 capsules which contain 180 mg EPA and 120 mg DHA. Omega-3 and placebo capsules were similar in weight, size, shape, taste, color, and odor. Placebo capsules were also produced by Karen Pharma & Food Supplement company and contain 600 mg oral glycerin. Study patients were informed how to use their supplements and they were weekly followed by phone calls. Any adverse events were reported.

### Clinical assessments

At the onset and end of the trial, body weight and height were measured to the nearest 0.1 kg and 0.1 cm using the Seca scale and stadiometer (Seca), respectively. The individuals were measured when they were barefoot and wearing light clothing. BMI for age Z-score was computed by dividing weight in kilograms by height in meters squared according to age in the curve standard using WHO growth standards. Dietary intake was assessed through 24-h food recall (two weekdays and one weekend) by an expert person. Recalls were completed at the beginning and end of the intervention by face-to-face interview (two weekdays and one weekend). We analyzed the information about dietary consumption by Nutritionist IV software. For estimating the physical activity level of participants, physical activity questionnaire for children (PAQ-C) and physical activity questionnaire for adolescents (PAQ-A), which were validated in Iran, were applied (22). The PAQ-C, appropriate for ages 8–14, provides a summary of physical activity scores (PASs) derived from 9 items, each scored on a 5-point scale. Also, the PAQ-A which is appropriate for ages 14–20, provides a summary of PAS derived from 8 items, each scored on a five-point scale. The levels of physical activity were reported in three categories including low activity, moderate activity, and high activity levels based on the PAQ-A scoring protocol (22, 23). Blood pressure was measured using a mercury sphygmomanometer after at least 5-min of resting. The measurement was performed on two occasions and the mean of the two was considered as the individual's blood pressure.

### Biochemical measurements

Blood samples (10 ml) were drawn following 12-h overnight fasting and centrifuged at 3,000 rpm for 5 min to extract serum samples pre- and post-intervention. The concentration of total cholesterol (TC), triglycerides (TG), and high-density lipoprotein cholesterol (HDL-C) was evaluated using commercial kits (DIALAB Inc. kit, Vienna, Austria) before and after the intervention. Serum LDL-C value was calculated using the Friedewald formula. The concentration of fasting serum insulin (SI) and fasting blood sugar (FBS) was measured using a commercial enzyme-linked immunosorbent assay (ELISA) kit (IBT, USA) and glucose oxidase method, respectively. Hemoglobin A1C (HbA1c) was assessed using a commercial kit (BioRex Inc., Tehran, Iran) by an auto-analyzer (Mindray auto hematology analyzer). Moreover, blood urea nitrogen (BUN) and creatinine were examined by the diacetyl monoxime method and enzymatic method, respectively. Erythrocyte sedimentation rate (ESR) was evaluated by the Westergren method using a special tube. In addition, serum high-sensitivity C-reactive protein (hs-CRP) level was assessed using an ELISA kit (Paadco Inc. kit, Tehran, Iran) pre- and postintervention. Also, the following formulas were used to determine the quantitative insulin sensitivity check index (QUICKI) and homeostatic model assessment for insulin resistance (HOMA-IR). Proteinuria was evaluated by albumin dipstick by collecting urine samples.

QUICKI: 1/[log (fasting insulin μU/ml) + log (fasting glucose mg/dl)].

HOMA-IR: [fasting insulin (μU/ml) × fasting glucose (mg/dl)]/405.

### FMD and CIMT assessment

Flow-mediated dilation (FMD) and carotid intima-media thickness (CIMT) were assessed by an expert radiologist. For evaluating FMD, after overnight fasting and 10 min rest, patients lay down in the supine position in a quiet, temperature-controlled room and put their right hand in a comfortable position on a surface for imaging the brachial artery. Ultrasound imaging of the brachial artery was done by Doppler ultrasound (Samsung Medison UGEO H60, Seoul, South Korea) and in a longitudinal section, 2 cm above of antecubital fossa. An anterior and posterior section were selected between the lumen and the vessel wall for clear imaging and after that, the diameter of the vessel was measured from the anterior part to the posterior part between Media and Adventitia. A baseline rest image was acquired and the arterial diameter was measured. For the second scan, the cuff of the sphygmomanometer was closed around the arm and a 5-min occlusion of at least 50 mmHg above the systolic pressure was applied to the vessel. Finally, 90 s after the cuff was opened, the second measurement was performed. The following formula was used to calculate FMD, where d1 is the baseline brachial artery diameter and d2 is the brachial artery diameter after 90 s of cuff release.

FMD: (d2 – d1) × 100/d1

To assess CIMT, the thickness of two inner layers of the Intima and Media artery were measured by Doppler ultrasound (Samsung Medison UGEO H60, Seoul, South Korea). The distance between the main edge of the first line and the main edge of the second line in the carotid artery was considered CIMT. To increase the accuracy, all measurements were repeated four times.

### Statistical analysis

Data entry, coding, security, and storage were checked. Statistical analysis of all data was done by SPSS statistical software (SPSS Inc., Chicago, IL, USA, version 24) and *p* < 0.05 were considered statistically significant. The Kolmogorov–Smirnov test was used for assessing the normality of the data. Quantitative and qualitative variables were presented as mean (standard deviation) or median (25th−75th percentile), and frequency (percentage), respectively. To examine differences in qualitative variables between omega-3 and placebo groups, we applied the chi-square test. We used the independent sample *t*-test or Mann–Whitney U*-*test to determine differences in numerical variables between the omega-3 and placebo groups. In addition, the aforementioned tests were used to compare between-group alterations in outcome variables. To do a within-group comparison, we used the paired-sample *t*-test or its non-parametric equivalent, the Wilcoxon test. A general linear model was applied to adjust the effects of confounding factors and baseline differences.

## Results

Fifty-one patients completed the study (*n* = 26 in the omega-3 and *n* = 25 in the placebo, group). Four patients in the omega-3 group and five patients in the placebo group discontinued the trial due to the COVID-19 pandemic and increased fear of contracting the disease during sampling and low compliance. Also, no side effects were reported during supplementation with omega-3 (Figure 1). The mean age of participants in the intervention group was 13.8 ± 2.3 years and in the placebo group was 12.9 ± 2.4 years (*p* = 0.164). The duration of diabetes in omega-3 and placebo groups were 7.6 ± 2.3 vs. 7.4 ± 2.8 years, respectively (*p* = 0.688). On the other hand, there were 14 boys (54%) and 12 girls (46%) in the omega-3 group and 15 boys (60%) and 10 girls (40%) in the placebo group (*p* = 0.657). Sixteen people in the intervention group and 13 people in the placebo group had low physical activity (PAS between 1 and 2.33) and on the other hand, 10 people in the intervention group and 12 people in the placebo group had moderate physical activity (PAS between 2.34 and 3.66) and none of the participants in the study had intense physical activity (PAS between 3.67 and 5) (*p* = 0.492). Following the analysis of the participants' food recall questionnaire, there was no significant difference in oral intake of vitamins and minerals affecting vascular function and structure, including vitamin A, vitamin C, beta-carotene, vitamin E, zinc, sodium, calcium, selenium, phosphorus, iron, potassium, and fatty acids between the two groups at baseline and the end of supplementation.

Table 1 shows the comparison of post-intervention energy intake, insulin infusion, and anthropometric indices between the 2 groups. After 12-week supplementation, height and weight increased significantly in both groups, and between the two groups this change was not significant; also, BMI Z-score did not change (*p* = 0.829). There were no significant differences in reported insulin infusion (*p* = 0.128) and dietary energy intake (*p* = 0.07) at baseline or the end of the study.

**Table 1:** Participant characteristics before and after supplementation with omega-3.

**Parameter**	**Time**	**Omega-3 (***N*** = 26)**	**Placebo (***N*** = 25)**	**Mean difference (95% CI)**	***P*** **-value**
**Weight (kg)**	Baseline	50.2 ± 14.2	45.3 ± 14.2		0.219^a^
	Endpoint	50.7 ± 13.7	45.9 ± 14	0.1 (−0.5 to 0.6)	0.82^b^
	Change	0.5 ± 1.1	0.6 ± 1.1	0.1 (−0.68 to 0.56)	0.846^a^
	P-within	0.025 ^c^	0.016 ^c^		
**Height (cm)**	Baseline	159.8 ± 13.6	153.6 ± 15.8		0.138^a^
	Endpoint	160.8 ± 13.1	155.2 ± 15.7	−0.4 (−1.4 to 0.4)	0.293^b^
	Change	1 ± 1	1.6 ± 2.1	−0.6 (−1.5 to 0.2)	0.175^a^
	P-within	<0.001 ^c^	0.001 ^c^		
**BMI (Z-score)**	Baseline	0.01 ± 0.71	0.08 ± 0.75		0.709^a^
	Endpoint	−0.01 ± 0.66	0.05 ± 0.67	0.01 (−0.08 to 0.1)	0.829^b^
	Change	−0.02 ± 0.18	−0.03 ± 0.18	0.02 (−0.08 to 0.12)	0.71^a^
	P-within	0.719 ^c^	0.397 ^c^		
**Calorie intake (kcal)**	Baseline	2,348 ± 670	2,328 ± 432		0.899^a^
	Endpoint	2,399 ± 576	2,269 ± 423	114 (−9 to 237)	0.07^b^
	Change	51 ± 265	−58 ± 215	110 (−26 to 246)	0.111^a^
	P-within	0.332 ^c^	0.187 ^c^		
**Insulin infusion (unit)**	Baseline	50.3 ± 18.1	52.1 ± 24		0.767^a^
	Endpoint	50.3 ± 18.1	53.1 ± 23.8	−1 (−2.4 to 0.3)	0.128^b^
	Change	0 ± 1.5	1 ± 3.1	−1 (−2.4 to 0.3)	0.134^a^
	P-within	1 ^c^	0.1 ^c^		

All data presented as mean ± SD. CI: confidence interval; BMI: body mass index.

a Independent t-test.

b General linear model adjusted for baseline differences between groups.

c Paired t-test.

There was a significant reduction about FMD (*p* = 0.003) in omega-3 compared with the placebo group after 12 weeks of supplementation, which remains statistically significant after adjustment for baseline differences between groups, age, sex, and TG changes (*p* = 0.006). However, no significant change in CIMT was observed following omega-3 supplementation (*p* = 0.33) (Table 2).

**Table 2:** Effect of omega-3 supplementation on endothelial function and vascular structure.

**Parameter**	**Time**	**Omega-3 (***N*** = 26)**	**Placebo (***N*** = 25)**	**Mean difference (95% CI)**	***P*** **-value**	***P*** **2**	***P*** **3**
**MD (%)**							
	Baseline	6.5 ± 3.6	8.2 ± 5		0.169 ^a^	
	Endpoint	9.6 ± 2.8	7.6 ± 3.4	2.5 (0.9 to 4.1)	0.003 ^b^	0.006	0.006
	Change	3.1 ± 4.2	−0.6 ± 4	3.7 (1.4 to 6)	0.002 ^a^	
	P-within	0.001 ^c^	0.452 ^c^			
**CIMT (mm)**							
	Baseline	0.443 ± 0.093	0.462 ± 0.069		0.415 ^a^	
	Endpoint	0.438 ± 0.094	0.464 ± 0.07	−0.009 (−0.025 to 0.01)	0.312 ^b^	0.229	0.33
	Change	−0.005 ± 0.036	0.003 ± 0.021	−0.007 (−0.024 to 0.01)	0.376 ^a^		
	P-within	0.522 ^c^	0.502 ^c^		

All data presented as mean ± SD. CI, confidence interval; FMD, flow-mediated dilation; CIMT, carotid intima-media thickness; mm, millimeter.

a Independent t-test.

b General linear model adjusted for baseline differences between groups.

c Paired t-test.

P2 General linear model adjusted for baseline differences between groups, age, and sex.

P3 General linear model adjusted for baseline differences between groups, age, sex, and triglycerides (TG) changes.

As shown in Table 3, TG decreased significantly at the end of the intervention, after adjusting for sex, age, and baseline differences between groups (*p* = 0.022). Hs-CRP decrease significantly in omega-3 group (*p* = 0.031); however, this change was not significant compared to the placebo group (*p* = 0.192). There was no significant change in FBS, HgA1c, serum insulin, and other metabolic indices between the two groups at the end of supplementation.

**Table 3:** Metabolic parameters before and after treatment with omega-3.

**Parameter**	**Time**	**Omega-3 (***N*** = 26)**	**Placebo (***N*** = 25)**	**Mean difference (95% CI)**	***P*** **-value**	***P*** **2**
**hs-CRP (mg/l)**						
	Baseline	2.6 ± 1.3	2.6 ± 1.3		0.981^a^	
	Endpoint	2.2 ± 1.1	2.5 ± 1.2	−0.2 (−0.6 to 0.1)	0.192^b^	0.221
	Change	−0.4 ± 0.8	−0.1 ± 0.8	−0.2 (−0.7 to 0.2)	0.257^a^	
	P-within	0.031^c^	0.45 ^c^			
**ESR (mm/hr)**						
	Baseline	13.6 ± 6.1	13.6 ± 6.2		0.994 ^a^	
	Endpoint	12.3 ± 5.2	13.5 ± 6.2	−1.2 (−3.1 to 0.7)	0.205 ^b^	0.337
	Change	−1.3 ± 4.1	−0.1 ± 3.1	−1.2 (−3.3 to 0.8)	0.238 ^a^	
	P-within	0.115 ^c^	0.901 ^c^			
**Creatinine (mg/dl)**						
	Baseline	0.7 ± 0.1	0.7 ± 0.1		0.203 ^a^	
	Endpoint	0.7 ± 0.1	0.7 ± 0.1	0 (−0.1 to 0.1)	0.531 ^b^	0.267
	Change	0 ± 0.1	0 ± 0.1	0 (−0.01 to 0.01)	0.744 ^a^	
	P-within	0.94 ^c^	0.573 ^c^			
**uACR (mg/g)**						
	Baseline	9 ± 3.03	9.4 ± 3.06		0.648 ^a^	
	Endpoint	9.47 ± 2.75	8.75 ± 2.32	0.93 (−0.19 to 2)	0.103 ^b^	0.11
	Change	0.43 ± 1.96	−0.65 ± 2.87	1.07 (−0.3 to 2.45)	0.124 ^a^	
	P-within	0.228 ^c^	0.271 ^c^			
**BUN (mg/dl)**						
	Baseline	16.6 ± 5.1	16.6 ± 4.6		0.964 ^a^	
	Endpoint	17 ± 5.2	16.2 ± 3.9	0.8 (−0.9 to 2.5)	0.355 ^b^	0.26
	Change	0.4 ± 3.5	−0.4 ± 3.1	0.8 (−1.1 to 2.7)	0.406 ^a^	
	P-within	0.59 ^c^	0.522 ^c^			
**FBS (mg/dl)**						
	Baseline	129 ± 39	126 ± 42		0.809 ^a^	
	Endpoint	127 ± 36	122 ± 33	3 (−12 to 18)	0.687 ^b^	0.631
	Change	−2 ± 37	−4 ± 29	2 (−17 to 21)	0.846 ^a^	
	P-within	0.781 ^c^	0.519 ^c^			
**SI (μIU/ml)**						
	Baseline	18.6 ± 14.4	19.7 ± 10.5		0.763 ^a^	
	Endpoint	19.2 ± 14.5	18 ± 8.2	1.8 (−3.5 to 7.2)	0.493 ^b^	0.741
	Change	0.6 ± 9	1.7 ± 12.8	2.3 (−3.8 to 8.5)	0.453 ^a^	
	P-within	0.715 ^c^	0.517 ^c^			
**QUICKI**						
	Baseline	0.31 ± 0.04	0.3 ± 0.04		0.476 ^a^	
	Endpoint	0.3 ± 0.03	0.3 ± 0.02	0 (−0.01 to 0.01)	0.999 ^b^	0.93
	Change	−0.01 ± 0.05	0 ± 0.03	0 (−0.02 to 0.02)	0.606 ^a^	
	P-within	0.504 ^c^	0.948 ^c^			
**HOMA-IR**						
	Baseline	5.7 ± 4.2	6.4 ± 4.9		0.558 ^a^	
	Endpoint	5.6 ± 3.5	5.4 ± 2.9	0.3 (−1.4 to 2.1)	0.679 ^b^	0.872
	Change	−0.1 ± 3.6	−1 ± 5.5	0.9 (−1.6 to 3.5)	0.474 ^a^	
	P-within	0.936 ^c^	0.375 ^c^			
**HbA1c (%)**						
	Baseline	6.8 ± 0.5	7 ± 0.3		0.252 ^a^	
	Endpoint	6.7 ± 0.4	7 ± 0.4	0.2 (−0.4 to 0)	0.111 ^b^	0.118
	Change	−0.1 ± 0.6	0 ± 0.5	−0.05 (−0.4 to 0.3)	0.723 ^a^	
	P-within	0.618 ^c^	0.972 ^c^			
**SBP (mmHg)**						
	Baseline	109 ± 8.1	106.4 ± 6.2		0.2 ^a^	
	Endpoint	107.7 ± 6.7	106.6 ± 5.3	−0.3 (−3 to 2.2)	0.772 ^b^	0.788
	Change	−1.3 ± 5.7	0.2 ± 5.3	0.5 (−1.9 to 3)	0.324 ^a^	
	P-within	0.244 ^c^	0.852 ^c^			
**DBP (mmHg)**						
	Baseline	64.4 ± 5.3	64 ± 5.9		0.79 ^a^	
	Endpoint	65 ± 6	64 ± 5	0.7 (−1.5 to 3)	0.533 ^b^	0.659
	Change	0.6 ± 4.3	0 ± 4.5	0.5 (−1.9 to 3)	0.645 ^a^	
	P-within	0.502 ^c^	1 ^c^			
**TG (mg/dl)**						
	Baseline	102.2 ± 27.4	117.3 ± 37.6		0.106 ^a^	
	Endpoint	94.8 ± 28.1	117.2 ± 36.1	−8.5 (−15.3 to −1.7)	0.015 ^b^	0.022
	Change	−7.4 ± 10.7	−0.1 ± 13.1	−7.3 (−14 to 0.5)	0.034 ^a^	
	P-within	0.002 ^c^	0.952 ^c^			
**Chol (mg/dl)**						
	Baseline	142.4 ± 18.5	156.1 ± 29.3		0.052 ^a^	
	Endpoint	146.1 ± 19.3	156.8 ± 29.6	0.2 (−7.8 to 8.1)	0.962 ^b^	0.881
	Change	3.7 ± 14	0.7 ± 14.5	2.9 (−5.1 to 11)	0.468 ^a^	
	P-within	0.193 ^c^	0.796 ^c^			
**LDL (mg/dl)**						
	Baseline	91.2 ± 24.3	100.3 ± 30		0.236 ^a^	
	Endpoint	95 ± 21.5	100.7 ± 28	1.8 (−4.3 to 7.8)	0.555 ^b^	0.532
	Change	3.8 ± 13.9	0.4 ± 8.4	3.4 (−3.1 to 9.9)	0.298 ^a^	
	P-within	0.176 ^c^	0.814 ^c^			
**HDL (mg/dl)**						
	Baseline	53.8 ± 12.6	53.1 ± 13.1		0.846 ^a^	
	Endpoint	54.1 ± 11.8	52.1 ± 10.8	1.5 (−1.7 to 4.7)	0.353 ^b^	0.531
	Change	0.3 ± 6.5	−1 ± 6.2	1.3 (−2.2 to 4.9)	0.461 ^a^	
	P-within	0.813 ^c^	0.419 ^c^			

All data presented as mean ± SD. CI, confidence interval; CRP, C-reactive protein; ESR, erythrocyte sedimentation rate; BUN, blood urea nitrogen; uACR; urine albumin-creatinine ratio; FBS, fasting blood sugar; SI, serum insulin; QUICKI, quantitative insulin-sensitivity check index; HOMA-IR, homeostatic model assessment of insulin resistance; HbA1c, hemoglobin A1c; SBP, systolic blood pressure; DBP, diastolic blood pressure; TG, triglycerides; Chol, cholesterol; LDL, low-density lipoprotein; HDL, high-density lipoprotein.

a Independent t-test.

b General linear model adjusted for baseline differences between groups.

c Paired t-test.

P2 General linear model adjusted for baseline differences between groups, age, and sex.

## Discussion

We demonstrated that daily supplementation with 600 mg/day omega-3 (containing 180 mg EPA and 120 mg DHA) for 12 weeks in adolescents with T1DM could significantly increase FMD. In addition, following supplementation TG was significantly decreased in the intervention group compared to the placebo group. No significant changes were seen between the two groups in terms of other metabolic parameters and CIMT.

It has previously been reported that FMD as a factor of vascular dysfunction assessment, is decreased in patients with diabetes (9). In this study, it has been demonstrated that a daily intake of 600 mg omega-3 could increase FMD in children and adolescents with T1DM. Many studies have reported increased levels of inflammatory factors in people with T1DM compared to people without diabetes which can cause vascular dysfunction (24). Moreover, previous studies have shown that consumption of omega-3 fatty acids increases the ratio of these fatty acids to arachidonic acid in the cell membrane phospholipids and reduces the production of pro-inflammatory eicosanoids (25). Also, mediators derived from omega-3 fatty acids such as resolvins have anti-inflammatory effects (26). On the other hand, omega-3 fatty acids cause phosphorylation of endothelial nitric oxide synthase (eNOS) and consequently increase the production of NO and finally improve vascular function through activating AMP-activated protein kinase (27). Also, Hsu et al. showed that omega-3 fatty acids can increase the bioavailability of nitric oxide and improve vascular response by reducing reactive oxygen species (ROS) (28). To date, only one study has been performed addressing the effect of omega-3 supplementation on FMD in children and adolescents in all populations, and the results were consistent with our study. Engler et al. (29) reported a significant increase in FMD, HDL, and LDL following 6 weeks of supplementation with 1.2 g/day DHA in children and adolescents with familial hypercholesterolemia (high risk for CVD in adulthood). However, several studies in adults have examined FMD and vascular function after omega-3 supplementation, with conflicting results. Mahoney et al. showed that 6 months of omega-3 supplementation with 3.3 g/day did not cause any significant change in FMD in 27 adults with T1DM (18). The results of the study by Stirban et al. (19) showed that supplementation with 2 g/day of omega-3 fatty acids for 6 weeks in adults with T2DM did not improve fasting FMD, but significantly reduced postprandial FMD. On the other hand, Wang et al. conducted a meta-analysis study on sixteen clinical trials demonstrating that omega-3 supplementation could significantly increase FMD, however, limitations such as the heterogeneous population and the different doses and duration of treatment affected these results (30). In addition to BMI and duration of diabetes, another reason for the differences between children and adults could be progressive vascular dysfunction following aging (31).

There was no significant change in CIMT (as markers for carotid atherosclerosis) following the 3 months omega-3 supplementation. In line with our study, Mahoney et al. reported that omega-3 supplementation for 6 months in patients with type 1 diabetes was not associated with a reduction in CIMT (18). However, Tomoya et al. showed that omega-3 supplementation for a longer period (2.1 years) in T2DM patients significantly reduces CIMT (32). Overall, omega-3 fatty acids appear to be able to improve the CIMT through their anti-inflammatory and antithrombotic effects (33), however, due to the relatively stable structure of the vascular wall, longer interventions are required to observe significant changes (34).

Following supplementation with omega-3, there was a significant reduction in TG levels in the intervention group compared to the placebo group. In line with our trial, Natto et al. revealed that administration of omega-3 polyunsaturated fatty acid (PUFAs) causes a significant reduction in TG in patients with diabetes (35). Similarly, in the study performed by Chauhan et al. omega-3 supplementation was effective in reducing TG levels in diabetic dyslipidemia (36). The exact mechanism by which omega-3 FAs had TG lowering effects returns to the high affinity of omega-3 FAs for peroxisome proliferator-activated receptor (PPAR) followed by enhancement of beta-oxidation and fatty acid metabolism. Moreover, omega-3 FAs may decrease hepatic TG synthesis through inhibition of acyl coA1 and diacylglycerol acyltransferase (37). Also, omega-3 FAs stimulate other nuclear receptors including hepatocyte nuclear factor 4α, liver X receptor, and farnesol X receptor, which modulates TG levels (38). Shinnakasu et al. reported that improving lipid profile and TG levels in patients with diabetes can improve FMD (39). It seems that one of the possible reasons that omega-3 supplementation improved FMD in the present study could be its effect on lowering serum triglyceride levels.

In our study, following omega-3 supplementation, hs-CRP levels were significantly reduced in the intervention group. Many systematic review and meta-analysis studies have also shown the effect of omega-3 PUFAs on lowering hs-CRP levels (40, 41). On the other hand, many studies have proven the effect of inflammation on vascular dysfunction (42). According to the reasons mentioned, another factor that can be effective in improving vascular function in our study is reducing inflammation. Also, other factors such as changes in blood pressure, BMI Z-score, and microalbuminuria could distort the results of the present study, none of which were significantly changed.

### Strengths and weaknesses

The strengths of the present study include using a randomized, double-blind, placebo-control design and also examining the effects of omega-3 supplementation on endothelial function, vascular structure, and metabolic parameters in adolescents with type 1 diabetes for the first time. Also, another strength of this study was the assessment of the effect of a dietary anti-inflammatory and antioxidant element with available food sources with low expected adverse effects, complementary to current medications. However, there are some weaknesses in this research. First, participants' self-reporting on dietary intakes and physical activity may influence our results. Second, no biomarker was considered for compliance of participants from omega-3 intake. Third, the small number of included subjects could affect the results. And finally, the duration of intervention could have been longer to influence study biomarkers, especially CIMT.

## Conclusion

In conclusion, daily consumption of 600 mg omega-3 fatty acid (containing 180 mg EPA and 120 mg DHA) in adolescents with T1DM for 12 weeks, significantly increased FMD along with a significant decrease in triglyceride. Following supplementation, the level of hs-CRP in the intervention group decreased but these changes were not significant compared with the placebo group and CIMT did not change. The present findings show that omega-3 intake can improve vascular function and may reduce the risk of CVD in adolescents with T1DM patients.

## Data availability statement

The original contributions presented in the study are included in the article/supplementary material, further inquiries can be directed to the corresponding author/s.

## Ethics statement

The studies involving human participants were reviewed and approved by Medical Ethics Committee of Iran University of Medical Sciences (IR.IUMS.REC.1400.070). Written informed consent to participate in this study was provided by the participants' legal guardian/next of kin.

## Author contributions

MKho: wrote the original draft, laboratory experiments, and data analysis. AS and NA: review and edit. BO, MA, and AH sampling. MS: data analysis. MKha: laboratory experiments. All authors read and approved the final manuscript.

## Funding

This research was supported by the Iran University of Medical Sciences (Grant number: 19738). Also, we would like to thank the Mofid Children's Hospital staff, Shahid Beheshti University of Medical Sciences, Tehran, Iran for their help.

## Conflict of interest

The authors declare that the research was conducted in the absence of any commercial or financial relationships that could be construed as a potential conflict of interest.

## Publisher's note

All claims expressed in this article are solely those of the authors and do not necessarily represent those of their affiliated organizations, or those of the publisher, the editors and the reviewers. Any product that may be evaluated in this article, or claim that may be made by its manufacturer, is not guaranteed or endorsed by the publisher.

## Abbreviations

T1DM: Type 1 diabetes mellitus
FMD: flow-mediated dilation
CIMT: carotid intima-media thickness
BUN: blood urea nitrogen
hs-CRP: high-sensitivity C-reactive protein
ESR: erythrocyte sedimentation rate
BMI: body mass index
TG: triglyceride
HDL-C: high-density lipoprotein cholesterol
LDL-C: low-density lipoprotein cholesterol
TC: total cholesterol
FBS: fasting blood sugar
HbA1C: hemoglobin A1C
HOMA-IR: hemostatic model assessment of insulin resistance
QUICKI: and quantitative insulin sensitivity check index
HO-1: heme oxygenase 1
GPx: glutathione peroxidase
NF-kB: nuclear factor-kB
PAQ-C: physical activity questionnaire for children
PAQ-A: physical activity questionnaire for adolescents
PAS: physical activity score
ELISA: enzyme-linked immunosorbent assay.
